# Combating *Spodoptera frugiperda* (Lepidoptera: Noctuidae) with Moringa-Synthesized Silica Nanoparticles and Its Combination with Some Insecticides

**DOI:** 10.1007/s13744-024-01210-0

**Published:** 2024-10-22

**Authors:** Amany D. Abd-Elnabi, Mohamed E. I. Badawy

**Affiliations:** 1 Cotton Leafworm Research Department, Plant Protection Research Institute. Agriculture Research Center, Giza, Egypt; 2 Department of Pesticide Chemistry and Technology, Faculty of Agriculture, Alexandria University, 21545-El-Shatby, Alexandria, Egypt

**Keywords:** Fall armyworm, Green synthesis, Silica nanoparticles, Plant extracts, Insecticide nanocarriers, *Moringa oleifera*

## Abstract

The fall armyworm (FAW), *Spodoptera frugiperda* (J. E. Smith), is a major agricultural pest known for developing resistance to insecticides. This study investigated a novel approach to manage FAW by silica nanoparticles (SiNPs) synthesized from eco-friendly leaf extract of *Moringa oleifera* Lam. (Moringaceae). This green synthesis method offers a sustainable and potentially safer alternative to traditional chemical processes. SiNP formation was confirmed by various techniques: UV–visible spectrophotometer, X-ray spectroscopy with energy-dispersive (EDX), scanning electron microscopy (SEM), and dynamic light scattering (DLS). The effectiveness of SiNPs alone and their combination with three common insecticides (emamectin benzoate, indoxacarb, and chlorpyrifos) were evaluated against third instar larvae of fall armyworm. While SiNPs after 24 h by leaf dipping method recorded limited insecticidal activity (LC_50_ = 9947.59 mg/L), it significantly enhanced the potency of all three insecticides. Combining SiNPs with emamectin benzoate resulted in the most dramatic increase in effectiveness compared to the insecticide alone with LC_50_ = 0.295 mg/L and 0.42 mg/L, respectively. This research suggests that moringa extract can be a valuable resource for the green synthesis of nanoparticles potentially useful in pest control. This approach could potentially reduce the amount of insecticide needed for effective pest control, leading to a more sustainable and environmentally friendly agricultural practice.

## Introduction

The fall armyworm (FAW), *Spodoptera frugiperda* (J. E. Smith) (Lepidoptera: Noctuidae), is a migratory polyphagous pest that is indigenous to tropical and subtropical regions of the Americas (Goergen et al. 2016). It can damage more than 350 species of plants belonging to 76 plant families such as maize, rice, sugarcane, wheat, barley, and sorghum (Montezano et al. 2018). Maize is the most preferred crop among all of them. According to Day et al. (2017), fall armyworm control is necessary to prevent maize yield losses ranging from 8.30 to 20.60 million tons per year (21–53% of overall production). In addition, about 70–80% of pesticides are applied ineffectively in the field, which might contaminate the environment through spray drift, surface runoff, and soil leaching (Fan et al. 2023).

In recent years, nanotechnology and nanoparticle synthesis have rapidly developed. There are a lot of significant applications for metal nanoparticles in the agriculture sector including fertilizers or as plant growth stimulants, nanopesticides (insecticides, herbicides, fungicides, pesticide carriers), and sensors (Pestovsky and Martínez-Antonio 2017). Loaded insecticides onto SiNPs increase the mortality rate against pests that infect the stored grain (Debnath et al. 2011; El-Naggar et al. 2020; Ziaee and Babamir-Satehi 2020). In addition, it could be used as mosquitos and *Spodoptera litura* (Fabricius, 1775) (Lepidoptera: Noctuidae) control agents (Barik et al. 2012; Baz et al. 2022; Debnath et al. 2012), as well as nanocarriers to minimize the environmental risks of insecticides (Yao et al. 2021). Also, SiNPs could be used as temperature-responsive nanocarriers for imidacloprid and abamectin for enhancing the toxicological properties against *Plutella xylostella* (Linnaeus) (Lepidoptera: Plutellidae) larvae and improving photolysis stability (Feng et al. 2020).

An enormous number of physical, chemical, biological, and hybrid procedures are usually used to prepare different types of nanoparticles (NPs) (Iravani et al. 2014). Most chemical methods are too expensive and also include the use of toxic and dangerous chemicals. In recent years, the increase of using a simple, green, and eco-friendly method was noticed to reduce the use of unsafe chemicals and produce nanoparticles less toxic and pure than those prepared by the chemical methods. The green methods depend on many natural resources such as plants, algae, microorganism extracts or/and metabolites, worms, actinomycetes, and waste products (Jadoun et al. 2021; Karande et al. 2021). Many researchers succeeded in green synthesis of various nanoparticles such as silica (Al-Azawi et al. 2019), selenium (Kalishwaralal et al. 2016), zinc oxide (Fakhari et al. 2019), silver (Awwad and Salem 2012), titanium oxide (Sundrarajan and Gowri 2011), copper oxides (Kumar et al. 2015), and gold (Elia et al. 2014).

Briefly, the green synthesis of NPs depends on the primary or secondary metabolites of natural products as reducing and capping agents for metal salt solution (precursor). The production of nanoparticles is naturally shown by a change in the color of the reaction solution, and it can change inorganic metal ions into metal NPs (El-Seedi et al. 2019). In the synthesis process of nanomaterials, metal ions in metal salt solution are recuperated from their salt precursors by the primary or secondary metabolites of natural products, which have reduction abilities. Then, the metal atoms merge to form metal NPs through the biological reduction of metal ions with various morphologies like cubes, spheres, rods, hexagons, and wires. In addition, the plant metabolites capped and stabilized NPs in stable morphology (Sajjad et al. 2018). The purpose of this work is to use *Moringa oleifera* Lam. (Moringaceae) leaf extract to produce silica nanoparticles sustainably. Evaluate the effectiveness of the biosynthesized SiNPs against the third instar larvae of the FAW in addition to studying the effects of the insecticides indoxacarb, emamectin benzoate, and chlorpyrifos both separately and in combination with the green-prepared SiNPs as a nanocarrier system.

## Materials and Methods

### Chemicals and Insecticides 

Tetraethoxysilane 98% (TEOS, (C_2_H_5_O)_4_Si) (FW = 208.33 g/mol) was obtained from Alfa Aesar (GmbH & Co. KG; Germany). Ethanol, acetone, methanol, sodium hydroxide, mercuric chloride, potassium iodide, sulfuric acid, hydrated copper(II) sulfate, gelatin, ferric chloride, and hydrochloric acid were obtained from El Gomhouria Company for Trading Chemicals, Egypt. In this study, distilled water was used and all used organic solvents were analytical grade. The insecticides used in this study were emamectin benzoate (Speedo®5.7% WDG Shoura Chemicals), chlorpyrifos (Pyrodan® 50% EC, the National Company for Fertilizers & Chemicals (Agrochem)), and indoxacarb (Avant® 15% EC, produced by FMC).

### Preparation of Hydroalcoholic Leaf Extract, Phytochemical Screening, and Analysis Using GC.MS

Dry leaves of *M. oleifera* (moringa) were purchased from the local herbal market, in Alexandria City, Egypt. For hydroalcoholic extraction, 20 g of moringa leaf powder was added to 100 mL of a mixture of distilled water and methanol (9:1) in a conical flask and stirred continuously for 1 h (50–65 °C and 500 rpm). The resulting extract was filtered using Whatman filter paper and kept at 4 °C till applied to other experiments. The standard methods provided by Harborne (1998) are used to analyze the phytochemical content of moringa leaves. Simple reactions based on color or precipitate formation changes were used to conduct the tests. The phytochemicals in the moringa plant that were studied included alkaloids, glycosides, flavonoids, phenols, saponins, and tannins.

Gas chromatography–mass spectrometry (GC–MS) (TRACE 1300 GC–MS ISQ (Thermo Scientific, Canada) is used to determine the molecular structure of a moringa extract. With a flow rate of one milliliter per minute, helium was used as a carrier gas. Initially, 45 °C was set for 2 min in the oven. Next, it was increased to 165 °C (4 °C/min) and finally to 280 °C (15 °C/min), with a post-run (off) at 280 °C. The GC/MS was given a Zebron capillary column, model number ZB-5MS, with an internal diameter of 30 m × 0.25 mm and a film thickness of 0.25 A. After 1 µL of the investigated extract was diluted in methanol (1:10), it was injected to assess and arrange its chemical structure. Electron impact ionization (EI) was carried out using the mass detector set to 250 °C and 70 eV, while the extract’s chemical composition was scanned and arranged using the mass spectrometer, which was moved from 50 and 500 m*/z*.

### Green Synthesis of Silica Nanoparticles (SiNPs)

**Fig. 1:**
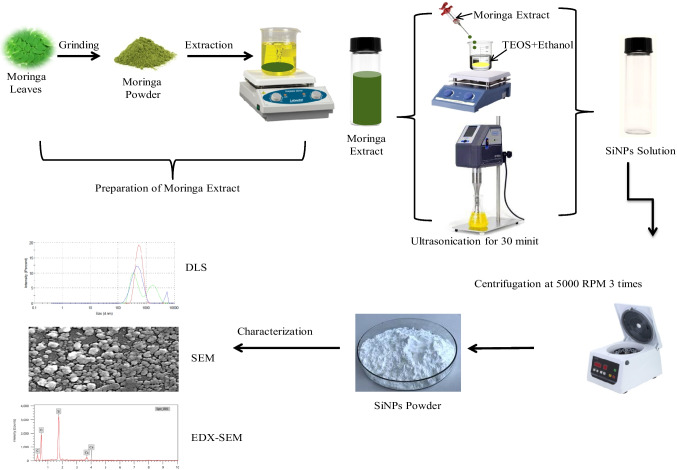
Schematic illustration of green synthesis of SiNPs

SiNPs were synthesized through the green synthesis method, as shown in Fig. 1, by adding 5 mL of moringa leaf extract dropwise into 10 mL of TEOS:ethanol:Tween 80 (1:1:0.01) solution as a precursor in a conical flask. The reaction was allowed for 24 h under continuous stirring at room temperature at 500 rpm. After that, the color of the mixture was changed to dark yellow. To finish the nanoparticle creation process 30 minutes of sonication and 15 minutes of 500 rpm stirring were required. The nanoparticle solution was purified by repeated centrifugation at 5000 rpm for 20 min followed by re-dispersion of the pellet in deionized water and ethanol. This process was repeated twice to isolate the pure SiNPs and exclude the presence of any unbound plant extract residue or TEOS. Then, it was placed in a hot air oven overnight at 100 °C. Finally, the white powder was obtained and stored in an airtight container till characterization and bioassay.

### Preparation of Loaded Insecticides/SiNPs

Insecticide loading was performed using the method of Wen et al. (2005) with some modifications. In a typical insecticide-loading process, 1% of silica nanoparticles and triple the LC_50_ dose of each insecticide are added to acetone. The insecticide/acetone mixture is stirred continuously by a magnetic stirrer at room temperature (26 ± 2 °C). A white turbid suspension appears, and the process continues for 60 min to ensure maximum drug loading.

### Characterization of SiNPs

Ultraviolet/visible spectrophotometer (UV/Vis, Alpha-1502, Laxco Inc, USA) was used to verify the success of the bioreduction of TEOS by hydroalcoholic extract of moringa into SiNPs; the obtained nanoparticles before drying were examined between the scan range of 390 to 700 nm. The nanoparticles absorb light at different wavelengths and are excited to give a broad peak due to the nanoparticle’s surface plasmon resonance nature in the reaction medium.

The morphology image and chemical composition of the green synthesized SiNPs were analyzed and photographed by a JEOL-scanning electron microscopy and energy-dispersive X-ray (EDX) spectroscopy coupled with a scanning electron microscope at the Faculty of Science, Alexandria University, Egypt.

The mean droplet size and PDI of silica nanoparticles were achieved by a dynamic light scattering method using Zetasizer Nano ZS (Malvern Instruments, UK) at room temperature. Nanosilica size was estimated by the average of three measurements and presented as mean diameter in nm (Tyagi et al. 2012).

### Fall Armyworm Rearing

FAW larvae were collected from maize fields in the Alnubaria region and transported to the laboratory for raring. In the incubator, the stock colony was reared on castor leaves under controlled conditions (25 ± 2 °C, 65 ± 5% RH, 14 L: 10 D photoperiod). The larvae were kept in a transparent plastic container (40 × 20 × 15 cm) until pupation. Pupae were kept in the same incubator until moths emerged. After exclusion, the moths were fed on 10% sucrose solution and left to lay eggs on pieces of paper, which were transported to a rearing container until the appropriate larval stage to examine the experiments (Dahi et al. 2020).

### Insecticidal Activity Assay Under Laboratory Conditions

Laboratory bioassays were conducted on the 3rd instar larvae of FAW using the leaf disc dipping method according to Idrees et al. (2023). Castor leaves were collected from unsprayed plants washed and air-dried. Serial doses of 0 (only water), 0.2, 0.5, 1, 10, 50, 100, 1000, 2000, 5000, 10,000, and 15,000 mg/L of each tested product, SiNPs, emamectin benzoate, indoxacarb, and chlorpyrifos, and their mixtures with 1% SiNPs were prepared. Leaf discs were dipped for 10 s in tested concentrations and allowed to dry at room temperature for 30 min. Leaf discs immersed in distilled water were labelled as control. Then, leaf discs were placed in individual Petri dishes (9 cm diameter). Each treatment (concentration) including control was replicated four times. The treatments were kept at a temperature of 25 ± 2 °C and 50–60 ± 5% RH. Larval mortality was recorded after 24 h of insecticidal exposure. The mortality was calculated and corrected by using Abbott’s formula (Abbott 1925).

### Statistical Analyses

The statistical program SPSS software, version 21.0 (SPSS, Chicago, IL, USA), was used for the statistical analysis. According to probit analysis, the log-dose response curves allowed for identifying the LC_50_ (concentration causing 50% of death) for the bioassays (Finney, 1971). By analyzing the relative growth rate (% control) against the logarithm of the compound concentration using least-square regression, it was possible to estimate the 95% confidence limits (CL) and standard error for the range of LC_50_ values for the compound assays on mortality. Abbott (1925) was used for the correction of natural mortality.

## Results

*Moringa oleifera* leaf extract was tested for the presence of phytochemicals such as saponins, flavonoids, alkaloids, glycosides, phenols, and tannins. The results of the qualitative phytochemical analysis of the moringa extract are shown in Table 1, revealing the presence of alkaloids, glycosides, flavonoids, phenols, and saponin. Fifty phyto-compounds were found using GC–MS analysis; the retention time, peak area percentage, and molecular weight are listed in Table 2. The principal substances that exhibit a high percent peak area are n-hexadecanoic acid (21.18), 9,12,15-octadecatrienoic acid (12.22), oxirane, tetradecyl (5.73), 1-Hexadecanol, 2-methyl (4.29), spirost-8-en-11-one 3-hydroxy, (3á, 5à, 14á, 20á, 22á, 25R), (3.65), genistin (2.77), 9,19-cyclolanostan-3-ol, 24, 24-epoxymethano acetate (2.22), and digitoxin (2.03).

**Table 1:** Phytochemical analysis of *Moringa oleifera* leaf

Phytochemical	Test used	Presence of phytochemical
Alkaloids	Mayer’s test	√
Flavonoids	Alkaline reagent test	√
Glycoside	Fehling’s test	√
Phenols	Ferric chloride test	√
Saponins	Frothing test	√
Tannins	Gelatin test	-

**Table 2:** GC–MS analysis of *Moringa olefera* extract

No	Compound name	Area %	Molecular formula	R.T
1	Oxirane, tetradecyl	5.73	C_16_H_32_O	21.38
2	n-Butylphosphonic acid	0.72	C_4_H_11_O_3_P	21.38
3	Oxirane, tetradecyl	1.50	C_16_H32O	22.24
4	n-Hexadecanoic acid	21.18	C16H_32_O_2_	24.61
5	n-Hexadecanoic acid	0.84	C_16_H_32_O_2_	24.73
6	Oxirane, tetradecyl	4.77	C_16_H_32_O	27.87
7	2-Hydroxy-(Z)9-pentadecenyl propanoate	1.04	C_18_H_34_O_3_	28.63
8	9,12,15-octadecatrienoic acid	12.22	C_18_H_30_O_2_	28.80
9	Octadecanoic acid	0.82	C_18_H_36_O_2_	29.36
10	Octahydropyrano[3,2-b]pyridin-6-one	1.29	C_8_H_13_NO_2_	36.24
11	Cyclohexane, 1,1′-dodecylidenebis[4-methy	0.95	C_26_H_50_	36.57
12	9,12,15-Octadecatrienoicacid	0.72	C_27_H_52_O_4_Si_2_	36.64
13	D-(-)-Fructose	2.47	C_6_H_12_O_6_	36.85
14	Octadecane,3-ethyl-5-(2-ethylbutyl)-	3.43	C_26_H_54_	39.96
15	1-Hexadecanol, 2-methyl	4.29	C_17_H_36_O	43.41
16	1-Monolinoleoylglycerol trimethylsilyl ether	1.08	C_27_H_54_O_4_Si_2_	45.71
17	1-Heptatriacotanol	1.22	C_37_H_76_O	46.03
18	6-Methyl-11-propenyl-5-(toluene-4-ulfonyloxy)-12,13-dioxatricyclo[7.3.1.0(1,6)]tridecane-8-carboxylicacid, methyl ester	0.67	C_24_H_32_O_7_S	46.13
19	Genistin	2.77	C_21_H_20_O_10_	46.94
20	Dihydroartemisinin,5-deshydroxy-6-deshydro	0.73	C_15_H_22_O_4_	47.42
21	2,7-Diphenyl-1,6-dioxopyridazino[4,5:2′,3′]pyrrolo[4′,5′-d]pyridazine	0.88	C_20_H_13_N_5_O_2_	47.45
22	9,10-Secocholesta-5,7,10(19)-triene-3,24,25-triol,(3á,5Z,7E)-	0.70	C_27_H_44_O_3_	47.88
23	9,12,15-Octadecatrienoic acid,2,3-bis[(trimethylsilyl)oxy]propyl ester, (Z,Z,Z)-	0.75	C_27_H_52_O_4_Si_2_	48.09
24	2-[4-methyl-6-(2,6,6-trime thylcyclohex-1-enyl)hexa-1,3,5-trienyl]cyclohex-1-en-1-carboxaldehyde	0.85	C_23_H_32_O	48.21
25	1,2-Propanediol,3-(hexadecyloxy)-,diacetate	0.69	C_23_H_44_O_5_	48.43
26	Rhodopin	0.79	C_40_H_58_O	48.54
27	1H-2,8a-Methanocyclopenta[a]cyclopropa[e]cyclodecen-11-one,1a,2,5,5a,6,9,10,10a-octahydro-5,5a,6-trihydroxy-1,4-bis(hydroxymethyl)-1,7,9-trimethyl-,[1S-(1à,1aà,2à,5á,5aá,6á,8aà,9à,10aà)]-	0.94	C_20_H_28_O_6_	48.79
28	9,12,15-Octadecatrienoic acid, 2,3-bis[(trimethylsilyl)oxy]propyl ester, (Z,Z,Z)	0.84	C_27_H_52_O_4_Si_2_	48.91
29	9,19-Cyclolanostan-3-ol,24,24-epoxymethano-,acetate	2.22	C_33_H_54_O_3_	49.06
30	Cucurbitacin B, dihydro	0.82	C_32_H_48_O_8_	49.18
31	10-Bromo-3,7,11-dimethyldodeca-2,3-dien-11-ol,1-acetoxy-	0.63	C_17_H_29_BrO_3_	49.23
32	á-Sitosterol	1.09	C_29_H_50_O	49.56
33	á-Sitosterol	1.42	C_29_H_50_O	49.59
34	9,12,15-Octadecatrienoic acid, 2-[(trimethylsilyl)oxy]-1-[[(trimethylsilyl)oxy]methy l]ethyl ester, (Z,Z,Z)	0.79	C_27_H_52_O_4_Si_2_	49.75
35	Spirost-8-en-11-one,3-hydroxy-,(3á,5à,14á,20á,22á,25R)	3.65	C_27_H_40_O_4_	49.88
36	t-Butyl-{2-[3-(2,2-dimethyl-6-methylene-cyclohexyl)-propyl]-[1,3]dithian-2-yl}-dimethyl-silane	0.80	C_22_H_42_S_2_Si	49.91
37	Withaferin A	0.90	C_28_H_38_O_6_	49.95
38	7-Hydroxy-6,9a-dimethyl-3-methylene-decahydro-azuleno[4,5-b]furan-2,9-dione	0.85	C_15_H_20_O_4_	50.11
39	9,12,15-Octadecatrienoic acid, 2,3-bis[(trimethylsilyl)oxy]propyl ester, (Z,Z,Z)	0.86	C_27_H_52_O_4_Si_2_	50.17
40	Betulin	0.88	C_30_H_50_O_2_	50.38
41	Octadecane, 1,1′-[1,3-propanediylbis(oxy)]bis	1.00	C_39_H_80_O_2_	50.48
42	Digitoxin	2.03	C_41_H_64_O_13_	50.56
43	9,12,15-Octadecatrienoic acid,2,3-bis[(trimethylsilyl)oxy]propyl ester, (Z,Z,Z)	0.83	C_27_H_52_O_4_Si_2_	50.62
44	1-Heptatriacotanol	0.70	C_37_H_76_O	50.74
45	Methyl 9,12-epithio-9,11-octadecanoate	0.92	C_19_H_32_O_2S_	50.82
46	Retinoyl-á-glucuronide6′,3′-lactone	1.22	C_26_H_34_O_7_	50.88
47	Rhodopin	1.29	C_40_H_58_O	50.90
48	Azafrin	0.80	C_27_H_38_O_4_	50.98
49	9,12,15-Octadecatrienoic acid,2,3-bis[(trimethylsilyl)oxy]propyl ester, (Z,Z,Z)-	0.71	C_27_H_52_O_4_Si_2_	51.44
50	Zeaxanthin	0.70	C_40_H_56_O_2_	51.91

**Fig. 2:**
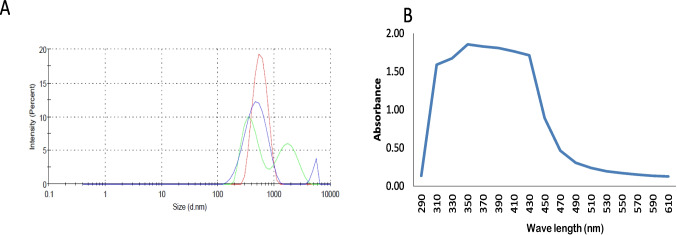
Droplet size distribution by DLS (**A**), and UV/Vis spectral analysis of the prepared green SiNPs (**B**)

The particle size of green silica nanoparticles was measured by DLS, the particle size was 529.5 nm, and the PDI was 0.075 as mentioned in Fig. 2 A. For verifying NP production, UV–Vis spectroscopic absorption was used at various wavelengths between 290 and 700 nm; the results showed a broad peak and this indicates the presence of SiNPs at 350–390 nm (Fig. 2 B).

**Fig. 3:**
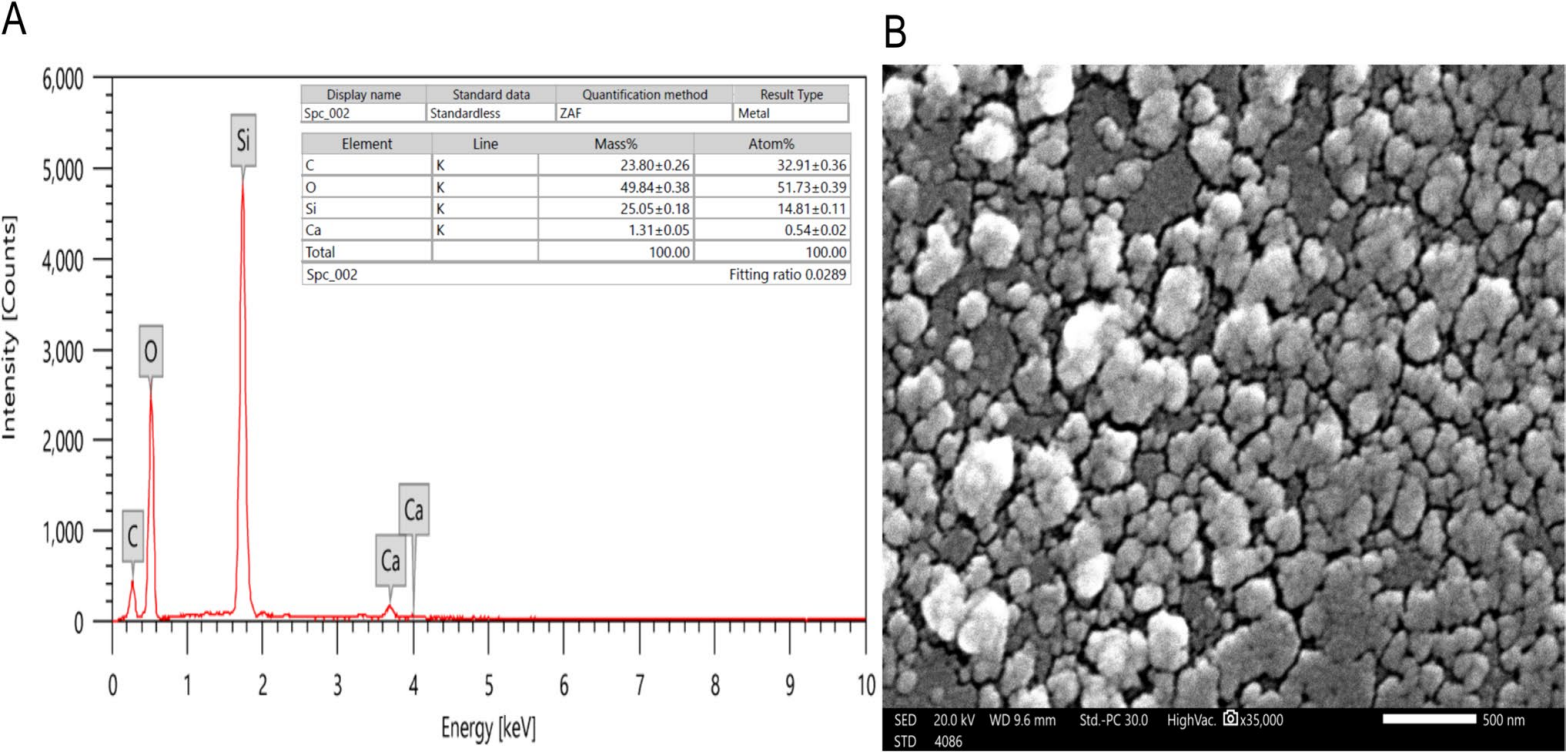
Energy-dispersive X-ray analysis (EDX) (**A**) and scanning electron microscopy (SEM) (**B**) images of green synthesized SiNPs

As shown in Fig. 3 B, SEM revealed that the SiNPs exhibited semi-spherical morphology. SEM combined with EDX was utilized to analyze the structure of silicon nanoparticles (SiNPs). The EDX results (Fig. 3 A) confirmed the presence of SiNPs and determined the elemental composition and purity of SiNPs mediated by moringa leaf extract. The main atomic percentages in green synthesized SiNPs are primarily C (23.80), Ca (1.31), O (49.84), and silica (25.05). The EDX spectra confirmed the successful formation of SiNPs with moringa leaf extract.

In preliminary lab tests, we evaluated the effectiveness of SiNPs, three insecticides, and their combination with 1% SiNPs against 3rd instar FAW larvae. Based on these preliminary trials, our results showed that all insecticides tested were effective against FAW. The data on larvicidal activity are shown in Table 3. The LC_50_ of SiNPs on 3rd instar FAW larvae after 24 h by leaf dipping recorded 9947.59 mg/L. The LC_50_ induced by emamectin benzoate is 0.42 mg/L, followed by indoxacarb and chlorpyrifos with LC_50_ = 967.47 and 1023.87 mg/L, respectively. The observed mortality rate in our study was high when the insect consumed food dipped in a mixture of pesticides and 1% SiNPs. The results showed that emamectin benzote + 1% SiNPs is the most promising formulation with LC_50_ = 0.295 mg/L, followed by indoxacarb and chlorpyrifos with LC_50_ = 481.12 mg/L and 615.16 mg/L, respectively.

**Table 3:** Toxicity effects of chlorpyrifos, emamectin benzoate, and indoxacarb and their mixture with 1% green SiNPs on *Spodoptera frugiperda* larvae under laboratory conditions after 24 h of treatment

Products	LC_50_^a^ (mg/L)	Confidence limits		Slope^b^ ± SE	Intercept^c^ ± SE	(*χ*^2^)^d^
		Lower	Upper			
SiNPs	9947.59	8469.94	12937.69	2.17 ± 0.371	8.67 ± 1.42	0.24
Chlorpyrifos 50% EC	1023.87	523.71	2897.53	0.79 ± 0.07	− 2.38 ± 0.19	11.08
Chlorpyrifos 50% EC + 1% SiNPs	615.16	249.03	2571.89	0.57 ± 0.05	− 1.59 ± 0.13	13.48
Emamectin benzoate 5% WP	0.42	0.32	0.53	1.40 ± 0.17	0.53 ± 0.08	0.72
Emamectin benzoate 5% WP + 1% SiNPs	0.295	0.005	0.65	1.36 ± 0.18	0.72 ± 0.08	4.33
Indoxacarb 15% EC	967.47	426.59	3785.99	0.53 ± 0.08	− 1.57 ± 0.15	4.76
Indoxacarb 15% EC + 1% SiNPs	481.12	229.12	1576.82	0.48 ± 0.0724	− 1.29 ± 0.14	3.07

*SiNPs* silica nanoparticles

^a^LC_50_ concentration causing 50% death for the larvae

^b^Slope of the concentration—mortality regression line ± standard error

^c^Intercept of the regression line ± SE

^d^Chi-square value

## Discussion

Silica nanoparticles have been successfully created using an easy and environmentally friendly process by using plant metabolite from moringa extract. It is commonly recognized that using plant extracts for the synthesis of NPs is a competitive and efficient process (Allafchian et al. 2016). Plant metabolites, such as terpenoids, polyphenols, sugars, alkaloids, phenolic acids, and proteins, play an essential role in the bioreduction and capping of metal particles to achieve the stability of the prepared metal nanoparticles (Makarov et al. 2014). It was noticed that moringa leaf extract could easily biosynthesize a wide variety of nanoparticles (Jadhav et al. 2022). Active ingredients found in moringa leaves function as stabilizing, reducing, and capping agents as well as producing biosynthesized metal nanoparticles (NPs). So many researchers used moringa extract in synthesizing different successful types of nanoparticles (Jadhav et al. 2022; Moodley et al. 2018). For instance, Shalaby et al. (2022) prepared FeO, NiO, MgO, CuO, Au, ZnO, Ag, and La_2_O_3_ nanoparticles by using moringa. Our findings are consistent with the research of Abd Rani et al. (2018) and Bagheri et al. (2020), who found that moringa contains a wide range of phyto-constituents, such as phenolic acids, glucosides, flavonoids, terpenes, alkaloids, saponins, and steroids (Makarov et al. 2014). In addition, Khalid et al. (2023) and Mensah et al. (2012) found similar consistency as we found such as flavonoids, phenols, alkaloids, saponin, and the absence of tannins.

Previous studies used GC–MS analysis for moringa leaf extract and found similar compounds to the present work which are fatty acids such as 9, 12, 15-octadecatrienoic acid and hexadecanoic acid are among the major components (Adeyemi et al. 2021; Khan et al. 2022; Syeda and Riazunnisa 2020).

The characterization of SiNPs was confirmed by many researchers using UV–Vis as a characterization tool and similar results were detected when SiNPs were produced from other resources (Babu et al. 2018; Djangang et al. 2015; Morales et al. 2019). The spherical shape of SiNPs using plant extract was confirmed by SEM (Periakaruppan et al. 2022; Sankareswaran et al. 2022). The EDX sharp peaks indicated that the synthesized SiNPs had a crystalline structure (Khan et al. 2017).

Numerous studies examined the use of various insecticides to combat FAW. Most research concurred with our findings which showed that emamectin, indoxacarb, and chlorpyrifos are effective against the FAW larvae. However, the median lethal concentration values varied, and this was due to variations in the population, bioassay method, and the instar of larvae.

For instance, Liu et al. (2022) studied the effect of emamectin benzoate on 3rd instar FAW larvae after 24 h and found that the LC_50_ was 0.106 mg/L. In addition, Amein et al. (2023) obtained LC_50_ values = 0.18 mg/L when the 4th instar FAW larvae were treated with emamectin benzoate. Ahissou et al. (2021) evaluated the susceptibility of 3rd instar larvae of FAW different populations to seven commercial insecticides in Burkina Faso. The authors found that emamectin benzoate was the most effective and the LC_50_ values were within the range of 0.00033–0.00038 mg/L.

Leaf-dipping bioassays method using 3rd instar larvae recorded LC_50_ values (0.11–0.12 ppm) for emamectin benzoate (Dileep Kumar and Murali Mohan 2022). Deshmukh et al. (2020) studied the effect of emamectin benzoate, indoxacarb, and other insecticides on the 2nd instar larvae by the leaf-dipping method; they found that emamectin benzoate showed the highest toxicity among all insecticides with LC_50_ = 0.005 mg/L, and indoxacarb demonstrated moderate effect with LC_50_ = 0.29 mg/L. The field applications also supported the same results.

Using the corn husk soaking as a bioassay method, three different populations of FAW were used to test the toxicity of emamectin benzoate and indoxacarb. The highest mortality rates were seen with emamectin benzoate (80:100%) when treated with 0.018 g/L, and indoxacarb ranged from 42:65% when treated with 0.047 g/L after 72 h (Bonni et al. 2020).

Suryani et al. (2022) examined the susceptibility of emamectin benzoate by mixing it with an artificial diet against 1st larvae of five different field populations and one laboratory population of FAW. After 7 days, the LC_50_ values ranged from 0.11 mg/L to 0.39 mg/L compared to the laboratory population (LC_50_ = 0.24 mg/L). However, the toxicity of chlorpyrifos on the 4th larval FAW instar recorded LC_50_ value = 470 mg/L (Salem et al. 2023). The effect of chlorpyrifos against the 3rd instar larvae by leaf dip bioassay recorded LC_50_ values within the range 199–377 mg/L (Dileep Kumar and Murali Mohan 2022) and 99.73–106.32 mg/L (Ahissou et al. 2021). Long-term usage of synthetic pesticides can harm crops and the environment and lead to insecticide resistance. Researchers are actively looking for options that can safely control this pest while being successful.

A family of nanomaterials known as nanocarriers can enable the targeted delivery and controlled release of fertilizers and insecticides in plants (Patra et al. 2018). Nanopesticides’ increased surface area to volume ratio and surface energy make it easier for an effective agent to penetrate and adhere to a plant’s surface. Therefore, the use of nanopesticides could significantly boost their efficacy. Silica nanoparticles offer a variety of benefits over bulk silicon sources for use in managing insect pests. SiNPs can act as a carrier for the pesticide to be delivered in a controlled release or as an insecticide to kill the targeted pest insects (Saw et al. 2023). These findings align with many other studies that have used SiNPs as insecticide carriers to boost their potency. It has been established that SiNP is a reliable and safe source of insecticides that may be employed at low and ecologically friendly dosages to control various other insect pests (Attia et al. 2023; Saw et al. 2023).

The residual toxicity of silica nanoparticles loaded with chlorpyrifos (Ch-SNPs) against adults of *Rhyzopertha dominica* (Fabricius, 1792) (Coleoptera: Bostrichidae) and *Tribolium confusum* (Jacquelin du Val, 1861) Coleoptera: Tenebrionidae was assessed by Satehi et al. (2018) as a nanocarrier. Ch-SiNPs were discovered to be successful in controlling both tested insect species. Both species exposed on Petri dishes treated with 0.01 g/m^2^ Ch-SiNPs had 100% mortality even after 6 h of exposure and 7 days following treatment.

Another study on stored grain insects by Ziaee and Babamir-Satehi (2020) found that the mortality rate of *Trogoderma granarium* (Everts, 1899) (Coleoptera: Dermestidae) larvae was greatly increased by the administration of loaded deltamethrin and chlorpyrifos insecticides in silica nanoparticles. Abamectin®1.8% and abamectin loaded on mesoporous silica nanoparticles (MSiNPs) were tested for their toxicological effects on *P. xylostella* 3rd instar larvae by Feng et al. (2021). Abamectin/MSNs exhibited a longer duration and control on *P. xylostella* with a lower survival rate of 30% compared to abamectin®1.8% which was its survival rate reached 93%. Indoxacarb-loaded nanoparticles were created by Bilal et al. (2020) and showed greater insecticidal activity against *P. xylostella* compared to indoxacarb technical at the same doses. Additionally, treatment with indoxacarb-loaded nanoparticles reduced the activity of detoxifying enzymes such as GST, CarE, and P450 in *P. xylostella*.

## Conclusion

Synthesis of nanoparticles using biological agents is eco-friendly, low-cost, and capable of producing at room temperature. In the present study, *moringa* leaf extract’s phytochemicals act as reducing and stabilizing agents. We have characterized the SiNPs by UV–vis, SEM, and EDX analysis. The UV–vis spectra confirm the formation of green synthesized SiNPs based on a surface plasmon resonance study. The EDX results determined the elemental analysis, particle stabilization, and zeta potential. SEM results revealed spherical and uniform-shaped silica nanoparticles. The *moringa-*synthesized SiNPs were potent in controlling and carrying agents for insecticides. The leaf phytochemicals of *moringa* are responsible for forming silica nanoparticles and exhibited potent biological activity against the fall armyworm. Nanotechnology will overcome the limits of traditional pesticides by increasing pesticide efficacy.
